# 阻断PAK1激酶活性对急性巨核细胞白血病细胞分化及凋亡的影响

**DOI:** 10.3760/cma.j.issn.0253-2727.2022.06.010

**Published:** 2022-06

**Authors:** 淑瑾 王, 春晴 王, 雪婷 胡, 翔茹 于, 春玲 付

**Affiliations:** 徐州医科大学血液病研究所、徐州医科大学附属医院血液科 221000 Blood Diseases Institute, Xuzhou Medical University; Department of Hematology, the Affiliated Hospital of Xuzhou Medical University, Xuzhou 221000, China

**Keywords:** 白血病，巨核细胞，急性, PAK1抑制剂, 生长停滞, 多倍化, 细胞凋亡, Leukemia, megakaryocytic, acute, PAK1 inhibitor, Growth arrest, Polyploidization, Apoptosis

## Abstract

**目的:**

探讨阻断p21蛋白激活激酶1（P21 Activated Kinase 1，PAK1）活性对急性巨核细胞白血病（AMKL）细胞株（CHRF和CMK）增殖、分化和细胞凋亡的影响。

**方法:**

采用细胞计数法检测PAK1抑制剂（IPA-3和G5555）作用前后AMKL细胞增殖及集落形成能力；应用流式细胞术检测PAK1抑制剂对AMKL细胞周期的影响；Western blot法检测细胞周期蛋白cyclin D1和细胞凋亡相关蛋白Cleaved caspase 3的表达；使用慢病毒介导的shRNA转染技术干扰AMKL细胞中PAK1的蛋白表达水平，应用流式细胞术检测敲低PAK1激酶的活性对AMKL细胞中多倍体DNA形成能力和细胞凋亡的影响。

**结果:**

PAK1抑制剂以剂量依赖性的方式抑制AMKL细胞的增殖并降低细胞集落形成能力，与对照组相比差异均有统计学意义（*P*值均<0.05）；PAK1抑制剂降低了S期AMKL细胞的百分比，Western blot法检测显示磷酸化PAK1及cyclin D1的表达水平显著下降（*P*值均<0.05）；PAK1抑制剂通过上调Cleaved caspase 3表达诱导AMKL细胞凋亡；PAK1抑制剂IPA-3和G5555分别显示出不同的增加巨核细胞中多倍体DNA含量的能力，仅有高浓度的IPA-3及低浓度的G5555可增加多倍体的巨核细胞的数目；敲低PAK1激酶活性，CHRF细胞多倍体DNA含量从14％增至22％，CMK细胞从8％增至16％，凋亡比例分别增至16％和12％（*P*<0.05）。

**结论:**

PAK1抑制剂显著诱导AMKL细胞生长停滞并促进AMKL细胞凋亡；敲低PAK1的表达促进多倍体DNA的形成并诱导AMKL细胞凋亡。抑制PAK1的活性可能是控制AMKL的有效方法。

## Abstract (en)

**Objective:**

To investigate the effect of blocking P21 activated kinase 1 (PAK1) activity on the proliferation, differentiation, and apoptosis of acute megakaryocytic leukemia (AMKL) cell lines (CHRF and CMK).

**Methods:**

Cell counts were used to detect the effects of PAK1 inhibitors (IPA-3 and G5555) on AMKL cell proliferation inhibition and colony formation, and flow cytometry was used to detect its effects on AMKL cell cycle. The effect of PAK1 inhibitor on the expression of cyclin D1 and apoptosis-related protein Cleaved caspase 3 was detected using Western blot, while interference with the protein expression level of PAK1 in AMKL cells was assessed using lentivirus-mediated shRNA transfection technology. Flow cytometry was used to detect the effects of knockdown of PAK1 kinase activity on the ability of polyploid DNA formation and cell apoptosis in AMKL cells.

**Results:**

PAK1 inhibitors inhibited the proliferation of AMKL cells in a dose-dependent manner and reduced the ability of cell colony formation, and the difference was statistically significant when compared with the control group (*P*<0.05). Moreover, they also reduced the percentage of AMKL cells in S phase, and Western blot detection showed that the expression levels of phosphorylated PAK1 and cyclin D1 decreased significantly. Finally, PAK1 inhibitors induced AMKL cell apoptosis by up-regulating Cleaved caspase 3 and showed different abilities to increase the content of polyploid DNA in megakaryocytes. Only high concentrations of IPA-3 and low doses of G5555 increased the number of polyploid megakaryocytes, while knockdown of PAK1 kinase activity promoted AMKL cell differentiation and increased the apoptosis rate.

**Conclusion:**

PAK1 inhibitor significantly arrests AMKL cell growth and promotes cell apoptosis. Knocking down the expression of PAK1 promotes the formation of polyploid DNA and induces AMKL cell apoptosis. The above findings indicate that inhibiting the activity of PAK1 may control AMKL effectively.

急性巨核细胞白血病（AMKL）是急性髓系白血病（AML）的一种亚型，其骨髓细胞学特征是未成熟的巨核细胞增多。最近的研究提出可针对未成熟的AMKL细胞进行诱导分化治疗[1]–[3]，如Aurora激酶A（AURKA）抑制剂（diMF或MLN8237）有效诱导6133/MPL和CMK巨核细胞的分化，并延长AMKL移植小鼠的存活期[2]。

p21蛋白激活激酶1（P21 Activated Kinase 1，PAK1）是丝氨酸/苏氨酸激酶PAK家族的成员，PAK1的激活促进了肝癌细胞的增殖、迁移和侵袭以及乳腺癌干细胞的形成，表明该激酶在肿瘤的发展中起着重要作用[4]–[6]。最近的研究显示PAK在巨核细胞中被激活[7]，AML中高水平表达的PAK1也支持PAK1抑制剂应用于骨髓疾病的治疗策略[8]–[9]。然而，尚不清楚PAK1是否参与调节巨核细胞增殖、分化和凋亡。

本研究中我们对数据库中的唐氏综合征（DS）、AMKL和AML患者的PAK1总mRNA表达水平进行对比分析，证明PAK1 mRNA在AMKL中被富集。通过实验发现AMKL细胞中PAK1的异常高表达可能是阻碍巨核细胞成熟的重要因素之一。进一步检测敲低PAK1激酶活性对AMKL细胞增殖、多倍化和细胞凋亡的影响。

## 材料与方法

1. 患者样本：mRNA微阵列表达来自Gene Expression Omnibus（www.ncbi.nlm.nih.gov/geo）。GSE4119数据集（https://www.ncbi.nlm.nih.gov/geo/query/acc.cgi?acc=GSE4119），包含33个AMKL样本、8个AML样本、8个DS同时伴有一过性骨髓增生性疾病样本。其中AML样本作为阳性参考样本，DS样本作为阴性参考样本。

2. 巨核细胞株、试剂和仪器：AMKL细胞株CHRF（CHRF-288-11，CVCL_A280，来自德国菌种保藏中心）和CMK（ACC392，来自德国菌种保藏中心）在含有10％胎牛血清的RPMI 1640培养基，37 °C、5％CO_2_培养箱中培养；293FT细胞来自美国菌种保藏中心；PolyJet™DNA转染试剂为美国SignaGen Laboratories公司产品；嘌呤霉素puro为北京Inovogen Tech公司产品，PAK1抑制剂（IPA-3：PAK1激活的变构抑制剂；G5555：ATP竞争性的PAK1/2抑制剂[10]–[11]）购自美国MCE公司；甲基纤维素为美国Sigma公司产品；Heochst 33342为美国Invitrigon公司产品；EdU试剂盒为KeyGen公司产品；AnnexinⅤ-FITC抗体为美国BD公司产品；CD41抗体（133916）为美国Biolegend公司产品；抗体PAK1（#2602）、P-PAK1（Thr423、#2601）、细胞周期蛋白D1（#2922）和Cleaved caspase 3（#9662）均购自美国CST公司；流式细胞仪为美国BD公司产品。

3. 在AMKL细胞系中通过shRNA敲除PAK1：通过invitrogen web设计了两对靶向PAK1的短发夹RNA（shRNA），然后构建成慢病毒载体pLVshRNA-EGFP puro（shCTRL：5′-TTCTCCGAACGTGTCACGT-3′；shPAK1#1：5′-GCTTCAGGCAGTGTATACT-3′；shPAK1#2：5′-GGGTTGTTATGGAATACTTGG-3′）。将上述2 µg shRNA质粒和1 µg包装质粒（PSPAX2和PMD2G质粒）与PolyJet™DNA转染试剂在室温孵育20 min，然后共转染至293FT细胞。72 h后，收集病毒上清液，加入CMK和CHRF细胞，经嘌呤霉素筛选2周后，构建稳定敲低PAK1的AMKL细胞系。

4. 细胞存活率检测：将CHRF和CMK细胞按照每孔约2×10^5^个细胞接种于12孔板中，然后用不同浓度的IPA-3（0、0.3、1、3、5、10、20、40 µmol/L）或G5555（0、0.01、0.03、0.1、1、3、9、20 µmol/L）处理48 h后，收集细胞进行锥虫蓝染色，对其中的活细胞进行计数，通过Graphpad Prism 6分析细胞存活率。

5. 细胞增殖和凋亡检测：将CHRF和CMK细胞按照每孔约2×10^5^个细胞接种于12孔板中，并用不同浓度的IPA-3（0、2、5、10 µmol/L）或G5555（0、0.03、0.1、1 µmol/L）处理24 h。将细胞与EdU在37 °C下孵育2 h后用IC固定液和透化缓冲液进行固定和透化，然后用500 µl Click-it Plus反应混合物室温避光的条件下孵育细胞30 min，再用DAPI标记核酸，然后用流式细胞仪检测细胞的增殖周期。

将接种培养的CHRF和CMK细胞用不同浓度的IPA-3（0、2、5、10 µmol/L）或G5555（0、0.03、0.1、1 µmol/L）处理48 h，感染shCtrl和shPAK1质粒的CHRF和CMK细胞正常培养48 h，根据手册用AnnexinⅤ-FITC抗体染色30 min后用流式细胞仪检测细胞凋亡。所有数据采集均在流式细胞仪上进行，并使用FlowJo7.6.1软件进行分析。

6. 集落形成能力检测：将500个CHRF和CMK细胞加入4 ml含甲基纤维素和60％血清的RPMI 1640培养基中，充分混匀，细胞用DMSO、IPA-3（0、2、5、10 µmol/L）或G5555（0、0.03、0.1、1 µmol/L）处理，在6孔板中培养。将孔板放在37 °C、5％CO_2_培养箱培养7 d，并在显微镜下计数菌落。

7. 流式细胞术检测多倍体含量：将经过PAK1抑制剂或shRNA处理后的细胞与Heochst 33342一起孵育2 h后，收集细胞并用PBS洗涤两次。然后将细胞用APC标记的CD41抗体在4 °C条件下染色30 min。在流式细胞仪上分析染色的细胞。倍数（N）由DNA含量确定。在本研究中，≥8 N被归类为高倍性细胞。

8. Western blot法检测蛋白表达：收集细胞并用含有蛋白酶和磷酸酶抑制剂混合物的RIPA缓冲液在冰上裂解30 min。提取出的蛋白质用10％的SDS凝胶分离（120 V 1 h）后以电转移法转移到NC膜上，用5％BSA液封闭1 h，将其与特定的一抗（cyclin D1、Cleaved caspase 3、PAK1、p-PAK1）一起孵育，然后加入二抗。使用ECL底物检测反应性蛋白质并通过ImageQuant LAS4000（美国GE公司产品）进行可视化。所有抗体均以1∶1000的比例稀释。

9. 统计学处理：使用GraphPad Prism 6软件进行数据统计分析。数据以*x*±*s*表示。两组数据的比较采用*t*检验，而多组间比较则通过单因素方差分析。所有实验均重复3次，*P*<0.05为差异有统计学意义。

## 结果

1. PAK1抑制剂对AMKL细胞增殖及集落形成能力的影响：本研究首先分析了DS、AMKL和AML患者的PAK1 mRNA富集情况，发现PAK1 mRNA水平在DS、AMKL和AML患者中逐渐升高，提示PAK1在巨核细胞或髓系白血病的发生发展中起关键作用。随后，选择两种直接靶向PAK1的小分子抑制剂，检测其对CHRF和CMK细胞增殖的影响。结果显示，PAK1抑制剂以剂量依赖性的方式抑制AMKL细胞的增殖，5 µmol/L的IPA-3显著抑制CHRF和CMK细胞的增殖，而0.03和1 µmol/L的G5555分别显著抑制CHRF和CMK细胞的增殖（图1A和B）。此外，与对照组相比较，5～20 µmol/L的IPA-3、0.1～3 µmol/L的G5555可分别抑制AMKL细胞的集落形成能力（图1C和D）。

**图1:**
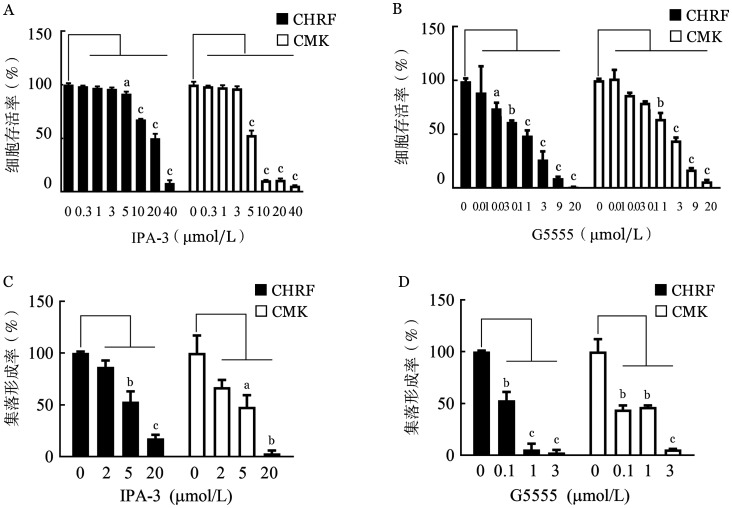
PAK1抑制剂IPA-3和G5555对CHRF和CMK细胞的增殖的影响（实验重复3次） A、B：IPA-3、G5555处理48 h的细胞存活率；C、D：细胞在含有IPA-3、G5555的甲基纤维素培养基中孵育7 d的细胞集落形成率。与对照组比较，^a^ *P*<0.05，^b^ *P*<0.01，^c^ *P*<0.001

2. PAK1抑制剂对AMKL细胞周期及细胞周期蛋白cyclin D1表达的影响：采用不同浓度的IPA-3或G5555处理AMKL细胞株24 h，并评估细胞周期阻滞情况。结果显示，处于S期的二倍体和多倍体细胞DNA被EdU成功标记，经PAK1抑制剂处理后，EdU阳性细胞所占比例显著降低。20 µmol/L IPA-3导致S期CHRF细胞比例从37％降至29％，S期CMK细胞比例从50％降至19％（图2A）。同样，3 µmol/L的G5555导致S期的CHRF细胞比例从37％下降到15％，但在同样处理下，G5555对CMK细胞周期的抑制作用有所减弱（图2B）。进一步Western blot分析显示，细胞经PAK1抑制剂处理后，磷酸化PAK1及cyclin D1的表达水平有所下降，且随着抑制剂浓度增加，该两种蛋白的表达水平显著下降（图3）。

**图2:**
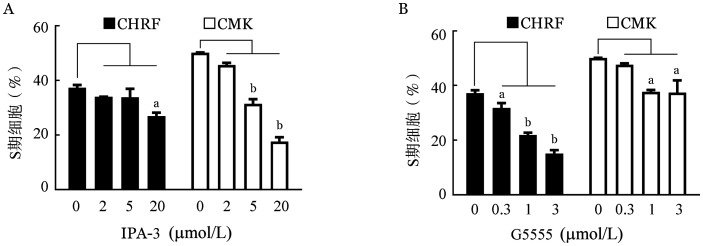
PAK1抑制剂IPA-3（A）和G5555（B）对CHRF和CMK细胞周期的影响（实验重复3次，与对照组比较，^a^ *P*<0.05，^b^ *P*<0.001）

**图3:**
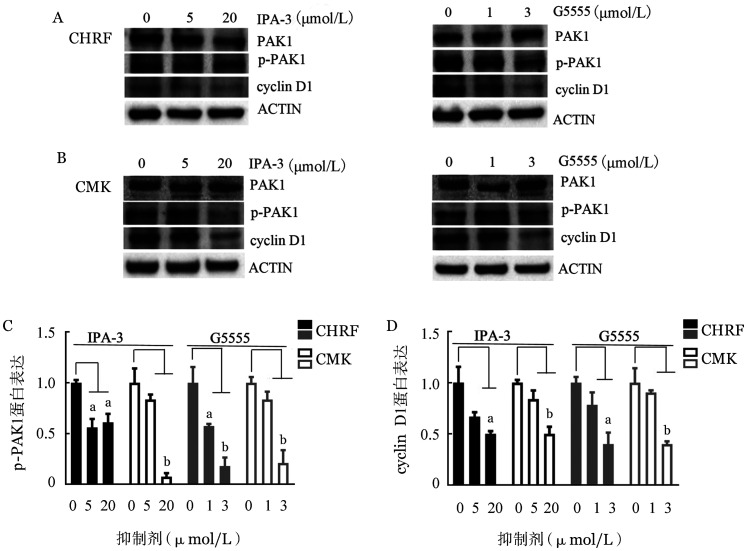
PAK1抑制剂IPA-3和G5555对CHRF和CMK细胞中PAK1及cyclin D1表达的影响（实验重复3次） A、B：Western blot分析PAK1抑制剂处理CHRF和CMK细胞24 h PAK1及cyclin D1表达；C、D：定量分析PAK1及cyclin D1的Western blot条带的灰度值。与对照组比较，^a^ *P*<0.05，^b^ *P*<0.01

3. PAK1抑制剂通过上调Cleaved caspase 3诱导AMKL细胞凋亡：采用不同浓度的IPA-3或G5555处理AMKL细胞株24 h，评估细胞凋亡情况。结果显示，20 µmol/L IPA-3处理后细胞凋亡率增加近一倍（图4A）。此外，3 µmol/L G5555处理也使CHRF细胞的凋亡率增加6.5％，CMK细胞的凋亡率增加2％（图4B）。Western blot结果提示，IPA-3和G5555均上调Cleaved caspase 3表达水平，其参与由PAK1介导的细胞凋亡（图4C和D）。

**图4:**
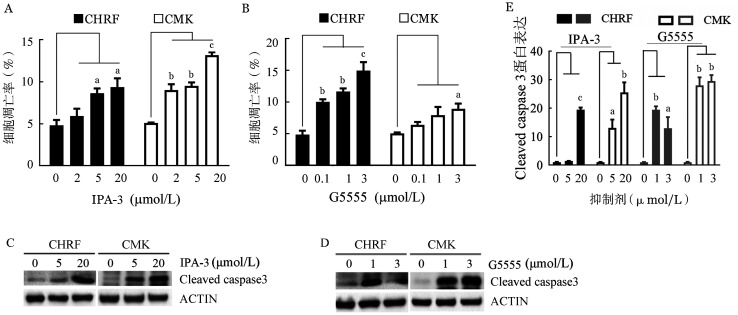
阻断PAK1激酶活性对CHRF和CMK细胞凋亡的影响（实验重复3次） A：IPA-3处理对细胞凋亡的影响；B：G5555处理对细胞凋亡的影响；C、D：Western blot检测IPA-3、G5555处理后Cleaved caspase 3的表达；E：定量分析Cleaved caspase 3/ACTIN的Western blot条带的灰度值。与对照组比较，^a^ *P*<0.05，^b^ *P*<0.01，^c^ *P*<0.001

4. 敲低PAK1对AMKL细胞分化、凋亡的影响：CHRF细胞经过IPA-3处理后，细胞表面分子CD41的表达水平以剂量依赖的方式显著增加，定量分析CD41阳性的平均荧光密度（MFI）显示，5 µmol/L的IPA-3作用后CD41分子的表达水平增加一倍以上；同样，低浓度的G5555（0.3 µmol/L）也显著提高AMKL细胞的CD41表达水平（图5A）。进一步对CD41阳性细胞核酸含量进行分析，结果显示，与CD41的表达水平相一致，两种PAK1抑制剂分别显示出不同的增加巨核细胞中多倍体DNA含量的能力（图5B）。此外，我们也对抑制剂诱导多倍体的巨核细胞的数目进行评估，如图5C所示，仅有高浓度的IPA-3及低浓度的G5555可增加多倍体的巨核细胞的数目。

**图5:**
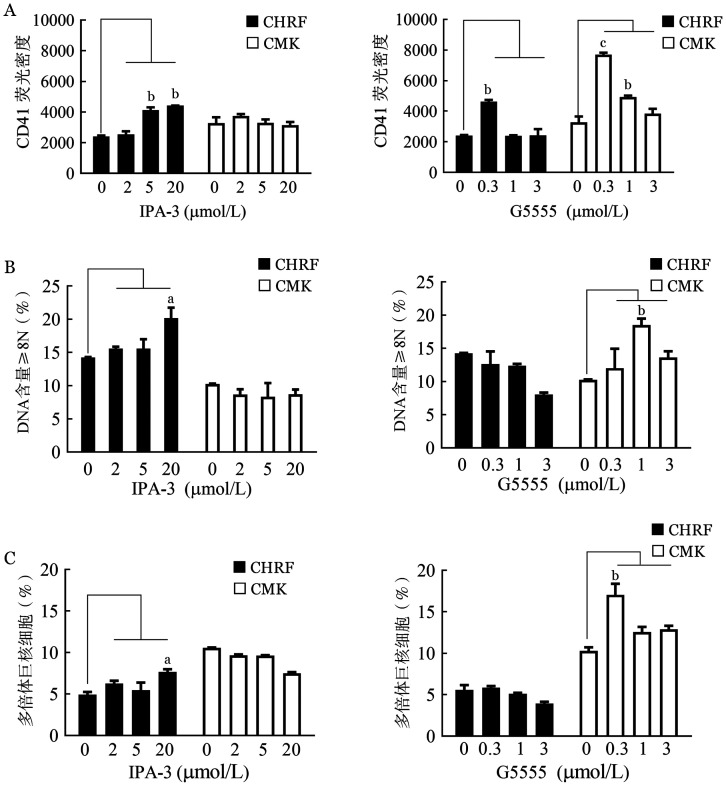
阻断PAK1激酶活性对CHRF和CMK细胞分化的影响（实验重复3次） A：PAK1抑制剂处理后CD41的表达水平；B：PAK1抑制剂处理后对多倍体DNA含量的影响；C：定量分析EdU阳性的AMKL细胞中核酸含量≥4N的巨核细胞的数目所占比例。N：倍数；与对照组比较，^a^ *P*<0.05，^b^ *P*<0.01，^c^ *P*<0.001

基于PAK1抑制剂对AMKL细胞表现出不同的诱导分化能力，本研究构建了特异性敲低PAK1的小干扰RNA，其中，转染shPAK1#2后CHRF和CMK细胞中PAK1表达水平下降约90％（图6A和B）。进一步分析显示，靶向敲低PAK1分别显著诱导了AMKL细胞的分化，使CHRF细胞中多倍体DNA含量从14％增加到22％，CMK细胞中从8％增加到16％（图6C和D）。此外，PAK1的敲低显著诱导细胞凋亡增加，CHRF和CMK细胞的凋亡比例分别增加至约16％和12％（图6E和F）。

**图6:**
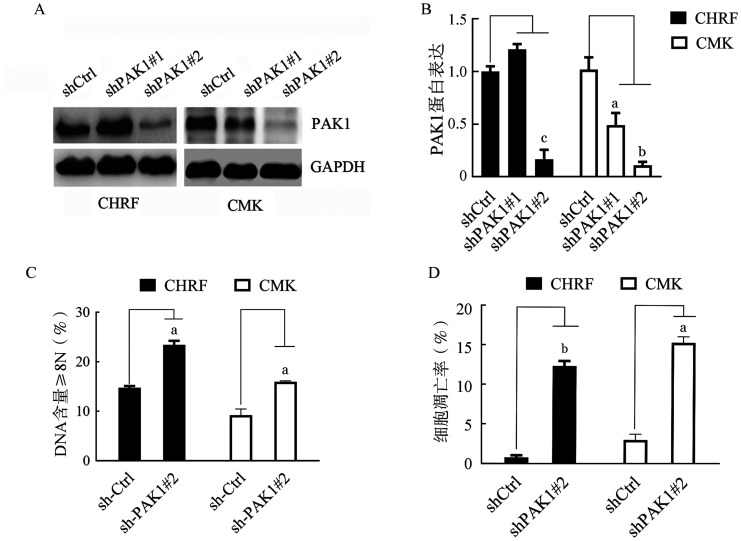
敲低PAK1后CHRF和CMK细胞的多倍体分布和凋亡分析（实验重复3次） A：Western blot检测PAK1的沉默效率；B：定量分析PAK1/ACTIN的Western blot条带的灰度值；C：流式细胞术分析感染shCtrl和shPAK1质粒的CHRF和CMK细胞的多倍体；D：用PE-Annexin Ⅴ染色流式细胞术检测感染shCtrl和shPAK1质粒的CHRF和CMK细胞凋亡。与对照组比较，^a^ *P*<0.05，^b^ *P*<0.01，^c^ *P*<0.001

## 讨论

AMKL是一种相对罕见的AML亚型，预后较差[12]。Aurora激酶抑制剂MLN8273通过诱导异常巨核细胞分化，有效缓解了JAK2或MPL基因突变的骨髓增殖性肿瘤（MPN）表型，Ⅰ期临床试验显示MPN患者缓解率为30％[13]。表明分化疗法是一种潜在的AMKL化疗方法。

在本研究中，PAK1 mRNA在AMKL和AML中的高富集表明其可能是促进髓系肿瘤形成的关键分子。巨核细胞成熟过程中经历两种有丝分裂：早期有丝分裂对祖细胞的增殖至关重要，晚期有丝分裂是细胞周期的一个独特阶段[14]–[15]。然而，大多数AMKL细胞仍处于早期增殖期，此时PAK1的高表达主要有助于细胞增殖，那么阻断PAK1的活性则为后期的有丝分裂阶段提供了机会。因此，本研究引入了PAK1抑制剂，评估它们对AMKL细胞系细胞增殖和分化的影响。尽管PAK1抑制剂（IPA-3和G5555）可以抑制CHRF和CMK细胞增殖和集落形成，但这些细胞系对PAK1抑制剂表现出不同的敏感性。IPA-3显著降低CMK细胞存活率及其S期DNA百分比，而G5555特异性抑制CHRF细胞的增殖。这些结果与在AML细胞系中观察到的结果相似[7]。推测CMK和CHRF细胞相对于这些PAK1抑制剂的不同敏感性可能是由于该两种细胞中所携带的突变基因不同。

本研究进一步发现，阻断PAK1激酶的活性可部分促进AMKL细胞的分化。高剂量的IPA-3显著增强CHRF细胞中EdU和Hoechst染色标记的多倍体巨核细胞（≥8N）形成的能力，但对CMK细胞没有类似的影响，这可能是由于IPA-3主要引起CMK细胞的生长抑制和凋亡。然而，低或中等剂量的G5555增加了CMK巨核细胞的多倍体率，高剂量的G5555反而产生了广泛的细胞致死性。值得肯定的是，特异性敲低PAK1的表达分别促进了CHRF和CMK细胞系的多倍化，提示PAK1的抑制剂可能具有潜在的其他作用靶点，从而表现出对AMKL细胞的不同诱导分化能力。

综上所述，PAK1 mRNA在AMKL中被显著富集，抑制PAK1激酶的活性延缓AMKL细胞的生长，部分促进AMKL细胞的多倍体化，最终诱导细胞凋亡。以上研究结果表明，PAK1可能是驱动AMKL增殖和分化的关键基因，抑制PAK1的异常活化可能是控制AMKL的有效方法。
